# Parotid mucoepidermoid carcinoma presenting as a cutaneous mass

**DOI:** 10.1016/j.jdcr.2026.04.063

**Published:** 2026-05-07

**Authors:** Asmita Acharya, Bijay Kunwar, Anup Ghimire, Sachchu Thapa, Ashima Kafle, Jagdish Kunwar, Amrit Panthi, Goma Dhami, Laila Lama Tangbetani

**Affiliations:** a Department of Dermatology, Venerology and Leprology, National Academy of Medical Sciences, Bir Hospital, Kathmandu, Nepal; b Tribhuvan University Institute of Medicine, Maharajgunj Medical Campus, Kathmandu, Nepal; c Department of Radiodiagnosis, National Academy of Medical Sciences, Bir Hospital, Kathmandu; d Nepal Medical College, Jorpati, Kathmandu, Nepal; e Department of Pediatrics, National Academy of Medical Sciences, Kanti Children’s Hospital, Kathmandu, Nepal; f Department of Orthopedics, National Academy of Medical Sciences, Bir Hospital, Kathmandu, Nepal

**Keywords:** cutaneous metastasis, diagnostic challenge, histopathology, mucoepidermoid carcinoma, parotid gland, palliative management, skin color

## Introduction

Mucoepidermoid carcinoma (MEC) is the most common malignant salivary gland neoplasm, accounting for roughly 10% to 30% of cases, predominantly arising in the parotid gland.^1^ Although MEC commonly remains confined to its primary site, it may rarely extend to the overlying skin or present as a cutaneous lesion, often mimicking primary skin tumors such as squamous cell carcinoma (SCC), basal cell carcinoma, or adnexal tumors.^2^^,^^3^ Cutaneous presentation of salivary MEC is exceedingly rare, accounting for less than 3% of cases.^4^ It frequently leads to diagnostic challenges due to clinical resemblance to more common cutaneous malignancies like basal cell carcinoma, or SCC.^5^

Accurate identification of cutaneous metastasis from MEC is vital, as management, prognosis, and therapeutic strategies differ significantly from those of primary cutaneous tumors.^6^ Histopathology, imaging, and when available, immunohistochemistry and molecular studies play crucial roles in confirming the diagnosis and determining the extent of disease.^7^^,^^8^ Herein we report a rare case of parotid gland MEC presenting as a mandibular skin lesion initially misdiagnosed clinically as cutaneous SCC, highlighting the significance of clinical awareness, histopathologic evaluation, and multidisciplinary management.

## Case reports

### Case history

A 67-year-old woman smoker presented with a progressively enlarging, indurated swelling over the left mandibular region for 2 years. The lesion was initially firm and nontender, slowly increasing in size. Six months before the presentation, it developed a friable, ulcerated component with intermittent bleeding, minimal discharge, and mild pain. No constitutional symptoms were reported.

**Fig 1:**
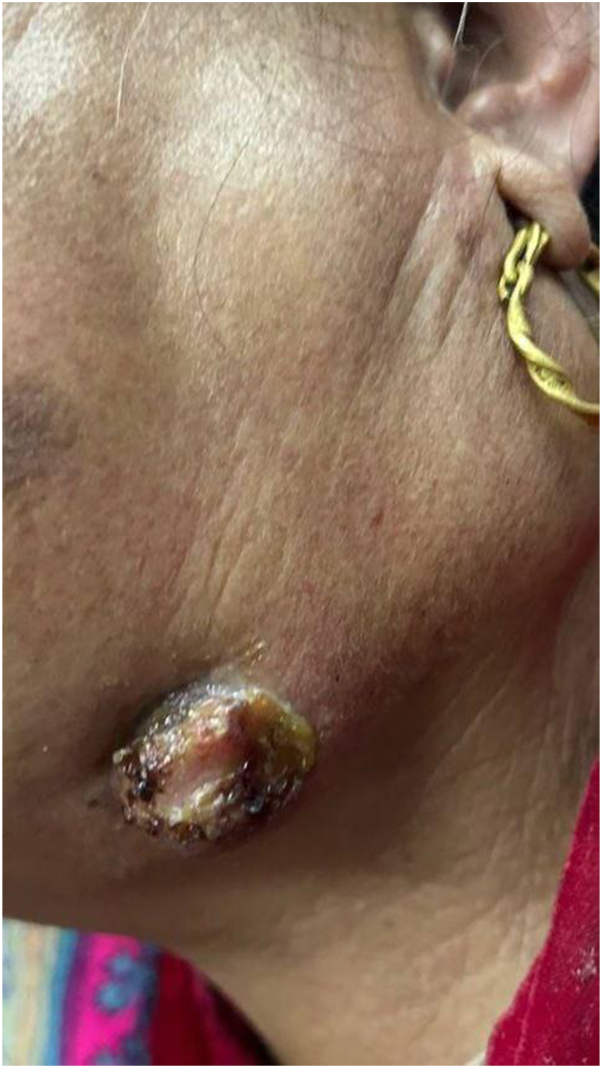
Ulcerated exophytic lesion measuring approximately 6 × 5 cm over the left mandibular region with irregular surface and ill-defined margins.

Dermatological examination revealed a 6 × 5 cm ulcerated indurated lesion with irregular raised margins, central necrosis, friability, and adherence to underlying structures (Fig 1). The surface was granular and crusted, clinically resembling a chronic nonhealing cutaneous malignancy. Based on the clinical appearance, a provisional diagnosis of cutaneous SCC was made, and an incisional biopsy was performed.

### Investigation and management

**Fig 2:**
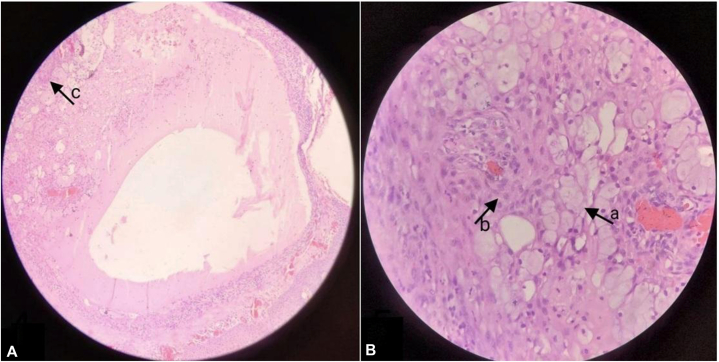
Histopathology showing dermis with cystic spaces containing abundant mucin-secreting cells (*a*), sheets of intermediate cells (*b*), and areas of squamous differentiation (*c*). **B**, High-power view demonstrating mucin-secreting cells along with intermediate cells features consistent with mucoepidermoid carcinoma. (**A** and **B,** original magnifications: **A,** ×100; **B,** ×400.)

Microscopic examination of the biopsy revealed dermal tissue with cystic structures lined by mucin-secreting cells, nests of intermediate cells, and focal squamous differentiation without keratin pearl formation (Fig 2, *A* and *B*). The tumor exhibited a mixture of mucinous, intermediate, and squamoid cells typical of MEC (Fig 2, *A* and *B*). These findings ruled out cutaneous SCC, favoring metastatic or locally invasive MEC.

**Fig 3:**
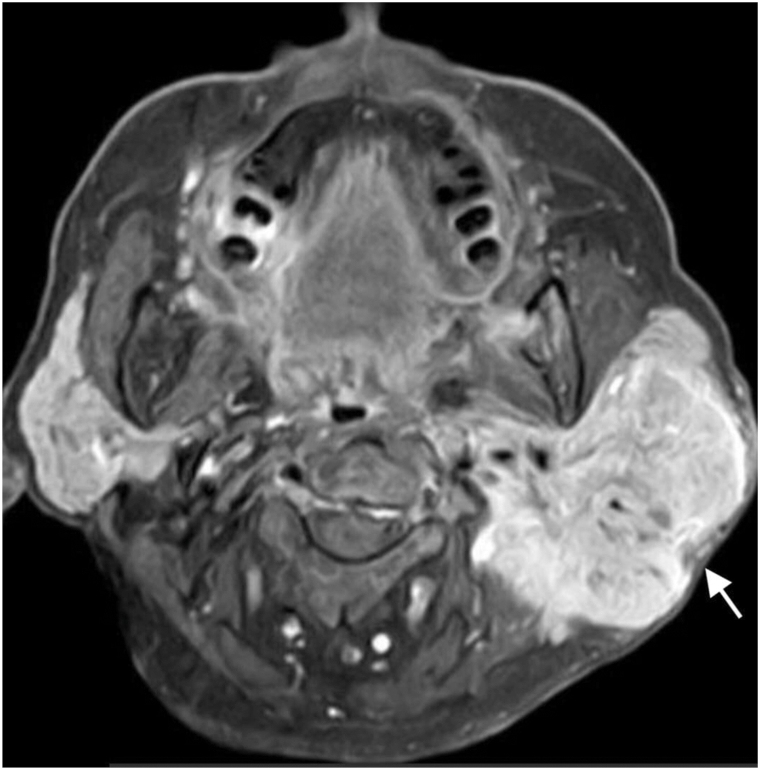
Contrast-enhanced axial magnetic resonance imaging neck at parotid level showing diffusely enlarged left parotid gland involving both lobes with T2 low signal, heterogeneous enhancement, cystic areas, and perineural spread (shown by *white arrow*). The lesion abuts the medial pterygoid muscle and mandible with medial parapharyngeal space compression, along with left cervical lymphadenopathy indicating locoregional malignant extension.

Contrast-enhanced magnetic resonance imaging of the neck revealed an enlarged left parotid gland with both superficial and deep lobe involvement, heterogeneous enhancement, cystic areas, and perineural spread. The mass abutted adjacent structures, including the mandible and medial pterygoid muscles, with evidence of ipsilateral submandibular lymphadenopathy (Fig 3). There was no evidence of distant metastasis. According to American Joint Committee on Cancer (8th edition) staging, the tumor was classified as stage IVA (T4a N1 M0), indicating locally advanced parotid MEC with regional extension and skin involvement. Given the advanced stage, extensive tissue invasion, and associated morbidity of radical surgical options, the patient opted against definitive curative treatment and was subsequently managed with palliative care.

## Discussion

Cutaneous spread of salivary MEC is rare but clinically significant, often indicating advanced disease.^8^^,^^9^ The most common primary source is the parotid gland, and metastatic involvement of the overlying skin occurs through direct extension, lymphatic, or hematogenous routes.^2^^,^^3^ In our case, the lesion was initially misinterpreted as cutaneous SCC because of its ulcerated, friable appearance and location.

The clinical presentation of skin or mucosal manifestations in MEC is highly variable, depending on tumor subtype, location, and histological features.^8^ Common findings include mass lesions, possible cystic or sclerotic changes, and variable symptoms based on tumor grade and stage.^8^ Differential diagnosis includes SCC, adenocarcinoma, melanoma, metastases from visceral MEC, and adnexal tumors.^4^ Metastatic MEC often mimics primary benign or malignant skin tumors, leading to misdiagnosis without histopathology. Mucin can be highlighted with periodic acid–Schiff or mucicarmine stains. Histologically, metastatic MEC shows mucin-secreting, squamous, and intermediate cells in cystic, solid, or papillary patterns.^10^ Unlike primary cutaneous MEC, metastatic lesions typically infiltrate the dermis without connection to epidermal adnexal structures.^1^^,^^9^ CK7, EMA, CEA, and mucin markers support MEC origin.^7^ CK20 negative, helps exclude gastrointestinal metastases, p63 strong and diffuse staining may favor primary cutaneous origin.^7^^,^^8^ Molecular markers such as *MAML2* gene rearrangement, commonly seen in salivary gland MEC, can also assist in confirming origin when available.^7^ In our case, histology confirmed MEC with a characteristic mix of mucin-secreting, squamous, and intermediate cells. Staging followed American Joint Committee on Cancer 8th edition salivary gland criteria. Although immunohistochemistry would have further supported the diagnosis, it was not performed due to strong clinical correlation and financial constraints.

Management of cutaneous metastatic MEC depends on disease extent/staging, tumor grade, and presence of systemic metastases. Surgical excision of solitary cutaneous lesions may be performed for local control.^6^ Chemoradiotherapy may be indicated in unresectable disease or when surgical margins are positive.^6^ Advanced tumors with perineural invasion or bony infiltration, as seen in this case, benefit from adjuvant radiotherapy to improve local control.^6^ This patient presented with a solitary lesion without evidence of distant metastatic disease (M0), and MRI demonstrated a locally invasive parotid tumor extending into adjacent soft tissues and the mandible, consistent with regional spread, which most likely represents direct contiguous extension of the primary parotid mucoepidermoid carcinoma to the overlying mandibular skin through adjacent perimandibular soft tissue planes, rather than true cutaneous metastasis. In contrast, true cutaneous metastases from salivary gland malignancies are rare and typically occur as multiple lesions in the setting of disseminated disease with a more favorable prognosis than cutaneous metastasis of primary salivary gland tumor (MEC) reflects advanced, sometimes incurable disease.^6^

High-grade MEC is associated with an elevated risk of recurrence and poor survival outcomes. Factors indicating poor prognosis include advanced age, high histologic grade, perineural invasion, lymph node involvement, and bone infiltration,^8^ all present in this case. Reported 10-year survival ranges from around 90% in low-grade tumors to 42% in high-grade MEC.^7^^,^^9^ For a T4a N1 M0 salivary gland carcinoma, an advanced staging, comorbidities, and personal preference resulted in opting for palliative care rather than aggressive multimodal treatment.^6^^,^^9^

## Conclusion

Cutaneous lesions over the head and neck region are not always primary skin tumors. They may represent underlying malignancies such as salivary gland MEC, particularly when presenting as progressive, atypical, or ulcerated nodules. This case underscores the importance of integrating clinical assessment with histopathology and imaging to differentiate primary cutaneous malignancies from metastatic disease. Timely diagnosis is essential to guide appropriate management and improve patient outcomes, especially in resource-limited settings.

## Conflicts of interest

None disclosed.
